# Investigation of Nurses' Assertiveness and Leadership Characteristics

**DOI:** 10.1002/nop2.70492

**Published:** 2026-04-08

**Authors:** Aybüke Çelik, Nazike Duruk

**Affiliations:** ^1^ Health Sciences Institute Eskişehir Osmangazi University Eskisehir Turkey; ^2^ Department of Nursing Eskisehir Osmangazi University Eskisehir Turkey

**Keywords:** assertiveness, assertiveness training, empowerment, healthcare communication, leadership, nursing, nursing education, nursing leadership

## Abstract

**Aim:**

This study aimed to examine the assertiveness levels and leadership characteristics of nurses.

**Design:**

This study employed a descriptive and cross‐sectional design.

**Methods:**

It was conducted in three hospitals within a province. The sample consisted of 367 nurses who voluntarily agreed to participate in the study. The data were collected between January 2021 and May 2022 using the Personal Information Form, the Leadership Orientations Scale and The Voltan‐Acar Assertiveness Inventory. Parametric tests were used for data analysis.

**Results:**

Among the nurses who participated in the study, 86.9% were female, 64.0% were under the age of 42, and 72.4% held a bachelor's degree. Those who attended scientific programmes, received assertiveness and leadership training, practiced their profession with passion and held a postgraduate degree had higher mean scores across all subdimensions of the Leadership Orientations Scale (*p* < 0.05). They also exhibited lower mean scores in the Shyness subdimension of the Voltan‐Acar Assertiveness Inventory than their counterparts (*p* < 0.05).

**Conclusion:**

Nurses who engaged in scientific programmes, received assertiveness and leadership training and dedicatedly practiced their profession exhibited stronger leadership qualities. As educational level increased, nurses were more capable of effectively utilising different leadership orientations. At the same time, they displayed higher levels of assertiveness.

**Relevance to Clinical Practice:**

Educational level, professional enthusiasm, having a nurse role model and participation in scientific programmes enhance assertiveness and leadership characteristics in nurses.

**Patient or Public Contribution:**

In today's evolving world, as in every profession, nursing requires modern leadership characteristics and assertive leaders. This study demonstrates the leadership characteristics and assertiveness levels of nurses, as well as how these traits are influenced by individual and professional factors. The findings are expected to contribute to the field.

## 1 Introduction

Assertiveness is a communication approach that involves expressing oneself openly, clearly and honestly while respecting both one's own and others' rights without belittling others. Assertiveness, also referred to as self‐confidence, includes the ability to say no when necessary, make requests, express emotions and demonstrate empathy (Voltan‐Acar and Öğretmen 2007). Assertiveness is a positive behavioural trait positioned between aggression and passivity. It is a skill that can be acquired and strengthened through repetition. Additionally, assertiveness varies based on individual characteristics and cultural influences. The ability to express oneself, engage in self‐improvement, defend one's rights, solve problems, cope with stress and make decisions is facilitated by assertive behaviour (Adana et al. 2009).

Assertiveness enables overcoming burnout by enhancing personal empowerment and constitutes an invaluable component in the practice of the nursing profession (Binuja and Nagarajaiah 2020). Assertive nurses are successful in collaborating with other healthcare professionals and establishing effective team relationships (Boone et al. 2008). This success is reflected in the prevention of medical errors (Manning 2006; McVanel and Morris 2010). Assertive nurses play a key role not only in preventing medical errors but also in improving the quality of nursing care (de Almeida Moura et al. 2020; Binuja and Nagarajaiah 2020). Furthermore, it has been reported that assertive nurses experience higher levels of job satisfaction and fulfillment, while also developing stronger leadership skills (Adana et al. 2009; Dinçer and Öztunç 2009).

A leader is an individual who benefits a society or institution, adapts to change and guides those around them towards common goals. A leader embodies ambition, self‐sacrifice, faith, determination and consistency while leading by example, staying motivated and inspiring others, actively listening and communicating effectively, demonstrating empathy, fostering innovation, making swift yet thoughtful decisions and possessing deep knowledge to ensure vision and execution align for success (Aksu and Yiğit 2019).

Leadership in nursing involves influencing individuals and their surroundings to unite towards a common goal. It is the effective utilisation of a nurse's knowledge and skills to fulfil shared objectives. In other words, leadership is the art of influencing others (Avcı and Başer 2019). To adapt to the evolving healthcare system, improve nursing practices, enhance patient care and fulfil professional roles, there is a need for nurses who demonstrate leadership behaviours such as self‐confidence, openness to communication, assertiveness and sensitivity (Kuşaklı Yılmaz and Bahçecik 2012). Nurses with leadership qualities emphasise teamwork, improve the quality of care and adopt a modern nursing approach (Avcı and Başer 2019; Özdemir Özkan et al. 2015). Work environments where nurse leaders are effective have been found to promote team engagement, increase job satisfaction, reduce work‐related stress and create a positive climate (Ardahan and Konal 2017). A study conducted with nurse managers highlighted that leaders should possess fundamental qualities such as teamwork, collaboration and knowledge sharing (Serinkan and İpekçi 2005). Leadership has introduced a new perspective to healthcare, fostering beneficial and developmental environments for both individuals and nurses. Ensuring the sustainability of these environments will only be possible by cultivating our own nurse leaders (Ardahan and Konal 2017). In recent years, the International Council of Nurses (International Council of Nurses [ICN] 2022) has emphasised the importance of leadership in nursing. In its 2017 theme, the ICN highlighted the significance of developing leadership in nursing to ensure nurses' participation in decision‐making processes. The World Health Organization (WHO) and ICN launched the ‘Nursing Now’ campaign in 2019, one of whose goals was to increase the visibility of nurses in leadership positions (World Health Organization 2020). Additionally, in 2019, the ICN underscored the importance of strengthening nurses' professional leadership skills through its theme ‘Nursing, Global Health, and Universal Health Coverage for All’ (ICN 2022).

In both national and international literature, studies assessing the assertiveness levels and leadership characteristics of nurses and nursing students were identified (Aksu and Yiğit 2019; Avcı and Başer 2019; Coşkun and Arslan 2021; Gürsoy and Uzun Aksoy 2021; Miles and Scott 2019). However, no research was found that examines nurses' assertiveness levels and leadership characteristics together. This study therefore aims to fill this gap by providing insights into the relationship between nurses' assertiveness levels and leadership characteristics.

The purpose of this study is to evaluate nurses' assertiveness levels and leadership characteristics.

### 1.1 Research Questions

- What are the assertiveness levels of nurses?
- Do nurses' individual and professional characteristics affect their assertiveness levels?
- What are the leadership characteristics of nurses?
- Do nurses' individual and professional characteristics affect their leadership characteristics?

## 2 Methods

### 2.1 Type of the Study

This study was conducted using a descriptive and cross‐sectional design.

### 2.2 Location of the Study

The data for the study were collected between 12 March 2021 and May 2022 using an online survey system. The study was conducted in a city hospital, a state hospital and a health practice and research hospital affiliated with the Provincial Health Directorate in a province.

### 2.3 Population and Sample

The study population consisted of 2484 nurses working at Eskişehir City Hospital, Yunus Emre State Hospital and Eskişehir Osmangazi University Health Practice and Research Hospital. The sample size was determined using a one‐way analysis of variance (ANOVA). A power analysis was conducted using G*Power 3.1.9.2, and based on a 95% power level and a 5% Type I error rate, it was found that at least 333 participants were needed. A simple random sampling method was employed, and the final sample comprised 367 nurses who voluntarily agreed to participate. Specifically, among the 981 nurses working at Eskişehir City Hospital, 228 participated; among the 750 nurses at Yunus Emre State Hospital, 76 participated; and among the 753 nurses at Eskişehir Osmangazi University Health Practice and Research Hospital, 63 nurses volunteered to take part in the study.

The study involved nurses employed at the institutions where the research was conducted. The participants were required to have unrestricted communication abilities, access to a smartphone or the internet and provide voluntary consent to participate.

### 2.4 Data Collection

After obtaining institutional permissions, meetings were held with the directors of nursing services at the three hospitals to obtain the email addresses of the nurse managers in the units where the research would be conducted. The data collection form, which was prepared online, was sent via email to the responsible nurse managers. In addition, the phone numbers of these nurse managers were obtained, and they were contacted by phone to ensure that all nurses had access to the survey. The completed surveys were automatically collected in the researcher's email inbox.

The research data were collected online using a Personal Information Form and scales. The survey link included an information page outlining the purpose and content of the study, as well as details about the researchers. The participants who selected the ‘I agree’ option on this page proceeded to participate in the study.

#### 2.4.1 Personal Information Form

This form was developed by the researchers based on the literature (Aksu and Yiğit 2019; Arabacı 2012). It consisted of 15 questions: five questions related to nurses' sociodemographic characteristics, five questions about their hospital and department, two questions regarding participation in courses, seminars and scientific programmes and three questions related to leadership.

#### 2.4.2 The Voltan‐Acar Assertiveness Inventory

The scale, developed by Voltan‐Acar and Öğretmen (2007), was used to assess assertive behaviour and measure individuals' levels of assertiveness. The terms assertiveness and self‐confidence are used interchangeably (Voltan‐Acar and Öğretmen 2007). The scale consists of 28 items and is structured as a six‐point Likert‐type scale. It has two subdimensions: assertiveness (11 items) and shyness (17 items). Each item is rated on a scale from 1 (does not describe me at all) to 6 (describes me very well). The total possible score ranges from 28 to 168: scores below 63 indicate highly shy individuals, scores between 64 and 98 indicate moderate shyness, scores between 99 and 133 indicate moderate assertiveness and scores of 134 and above indicate highly assertive individuals. The Cronbach's alpha reliability coefficients were reported as 0.830 for the shyness subdimension, 0.780 for the assertiveness subdimension, and 0.870 for the overall scale (Voltan‐Acar and Öğretmen 2007). In this study, reliability analysis demonstrated that the Voltan‐Acar Assertiveness Inventory had high reliability (*α* = 0.767), with the shyness subdimension (*α* = 0.902) and the assertiveness subdimension (*α* = 0.806) exhibiting strong reliability.

#### 2.4.3 Leadership Orientations Scale

The Leadership Orientations Scale (LOS) was used to determine leadership orientations and leadership styles. The scale was developed by Bolman and Deal (1990) to examine individuals' leadership orientations and was adapted into Turkish by Dereli (2003). The scale consists of three sections. The first section assesses leadership behaviours, the second evaluates leadership style, and the third examines managers' attitudes as both administrators and leaders. The scale comprises a total of 40 items. The first section, which evaluates leadership behaviours, consists of 32 items measured on a five‐point Likert scale. These items are categorised into four key subdimensions: Structural (Items 1, 5, 9, 13, 17, 21, 25, 29), Human Resource (Items 2, 6, 10, 14, 18, 22, 26, 30), Political (Items 3, 7, 11, 15, 19, 23, 27, 31) and Symbolic (Items 4, 8, 12, 16, 20, 24, 28, 32). Each subdimension consists of eight items. The Cronbach's alpha reliability coefficients for the subdimensions were reported to range between 0.84 and 0.88 (Dereli 2003). Each item is scored as follows: Never = 1, Rarely = 2, Sometimes = 3, Often = 4 and Always = 5. The total possible score for each subdimension ranges from 8 to 40, with higher scores indicating a higher tendency to exhibit leadership behaviours and lower scores indicating a lack of leadership behaviours. The maximum possible total score on the scale is 160 (Dereli 2003). Since the third section of the scale pertains to responsible nurses, it was not used in this study. Previous research utilised the first section of the LOS in studies aligning with the purpose of this research (Bayrakdaroğlu et al. 2020). In the reliability analysis conducted for this study, the LOS was found to be highly reliable (*α* = 0.945). Among its subdimensions, Human Resource Leadership (*α* = 0.816), Political Leadership (*α* = 0.848) and Symbolic Leadership (*α* = 0.880) were determined to have high reliability, while Structural Leadership (*α* = 0.764) was found to be considerably reliable.

### 2.5 Data Analysis

For data analysis, frequency distributions were used for categorical variables, while descriptive statistics (Mean ± SD) were provided for numerical variables. To determine the appropriate statistical tests, the Kolmogorov–Smirnov normality test (*n* > 50) was applied to all variables. The results indicated that the variables followed a normal distribution; therefore, parametric tests were used for comparisons. The independent samples *t*‐test was applied to examine differences between two independent groups, while One‐Way Analysis of Variance (ANOVA) was used for comparisons among more than two independent groups. In cases where significant differences were detected, Tukey's test was conducted to determine which groups differed from each other. The Pearson correlation coefficient was used to assess the degree of non‐causal relationships between two numerical variables. A *p*‐value of < 0.05 was considered statistically significant. The variables years of experience in the profession, the department in which nurses worked and the number of patients per nurse in the department were found to be statistically insignificant in the analysis and were therefore not included in Table 3.

### 2.6 Ethical Considerations

For the city and state hospitals, approval was obtained from the Provincial Health Directorate (15 March 2021). Institutional approval for the Health Practice and Research Hospital was granted by the Hospital Directorate (16 November 2020). Additionally, ethical approval was obtained from the Non‐Interventional Clinical Research Ethics Committee of Eskişehir Osmangazi University (dated 16 October 2020).

Permission for the use of the scales in the study was also obtained from the original authors. Participation in the study was voluntary, and an informed consent form was included on the first page of the online survey. The statement ‘I voluntarily agree to participate in this study, understand that I can withdraw at any time, and consent to the use of my data for scientific purposes’ was provided. Participants who did not check this box were unable to proceed to the questionnaire section. The informed consent form also included details regarding the purpose of the study, a brief explanation of the scales used, the number of questions to be answered, and the estimated time required to complete the survey.

Data collection was conducted in accordance with the Declaration of Helsinki, and research integrity and publication ethics were upheld throughout the study.

## 3 Findings

Among the nurses who participated in the study, 86.9% were female, 64.0% were under the age of 42, 59.7% were married and 80.9% resided in the city centre (Table 1). Additionally, 72.4% held a bachelor's degree. In terms of professional experience, 46.3% had 16 or more years of experience and 33.5% worked in internal medicine departments. The highest proportion of nurses (56.2%) reported caring for 5–10 patients per nurse. Regarding professional satisfaction, 71.9% stated that they enjoyed their profession, while 66.2% indicated that if given the chance, they would not choose nursing again. Moreover, 56.7% of the nurses reported attending scientific events such as congresses and symposiums, while 78.5% stated that they had not received any assertiveness or leadership training. When asked ‘What qualities should a nurse leader possess?’, responses were categorised based on the nurses' initial answers into knowledge level, fairness, assertiveness, dedication, empathy and team spirit. Among these, knowledge level and fairness were identified as the most critical attributes of a nurse leader, with the highest proportion (27.8%). Additionally, 61.6% of nurses stated that they did not have a nurse role model as a leader, while 55.0% considered themselves as leaders (Table 1).

**TABLE 1:** Distribution of nurses' demographic and professional characteristics (*n* = 367).

Variables	*n* = 367	%
Gender		
Female	319	86.9
Male	48	13.1
Age (Mean ± SD)	35.76 ± 8.877 years	
29 years and below	123	33.5
30–41 years	112	30.5
42 years and above	132	36.0
Marital status		
Married	219	59.7
Single	148	40.3
Longest place of residence		
City centre	297	80.9
District	61	16.6
Village	9	2.5
Educational level		
Vocational health high school	20	5.4
Associate degree	39	10.6
Bachelor's degree	266	72.4
Postgraduate degree	42	11.4
Years of professional experience (years)		
5.5 years and below	66	18.0
6–10 years	68	18.5
11–15 years	63	17.2
16 years and above	170	46.3
Department of employment		
Internal medicine units	123	33.5
Surgical units	60	16.3
Outpatient clinics	45	12.3
COVID unit	28	7.6
Paediatrics unit	14	3.8
Intensive care unit	74	20.2
Emergency unit	23	6.3
Number of patients per nurse in the department		
1–4 patients	42	11.5
5–10 patients	205	56.2
10 or more patients	118	32.3
Enjoyment of the nursing profession		
Yes	264	71.9
No	103	28.1
Willingness to choose the nursing profession again		
Yes	124	33.8
No	243	66.2
Participation in scientific programmes (Congress, symposium, workshop, etc.)		
Yes	208	56.7
No	159	43.3
Receiving assertiveness and leadership training		
Yes	79	21.5
No	288	78.5
Characteristics required in a leader nurse^a^		
Knowledge level	102	27.8
Fairness	102	27.8
Assertiveness	52	14.2
Dedication	38	10.3
Empathy	29	7.9
Team spirit	25	6.8
No response	19	5.2
Having a nurse as a leadership role model		
Yes	141	38.4
No	226	61.6
Perceiving oneself as a leader		
Yes	202	55.0
No	165	45.0

^a^ *n* = expanded.

Regarding the distribution of the Voltan‐Acar Assertiveness Inventory (VAAI) scores based on the participating nurses' demographic and professional characteristics, the mean shyness subdimension score was lower in women compared to men (*p* = 0.043). The mean assertiveness subdimension score was lower among those aged 29 and below compared to the other age groups (*p* = 0.017), with the difference being attributed to the 29 and below age group. The nurses residing in the city centre had lower total VAAI scores and lower shyness subdimension scores compared to those living in districts or villages (*p* = 0.019; *p* = 0.024, respectively). The nurses who enjoyed their profession had lower shyness scores than those who did not (*p* = 0.009). Additionally, the nurses who would choose the nursing profession again had higher assertiveness scores than those who would not (*p* = 0.028) (Table 3).

The nurses who participated in scientific programmes had higher assertiveness subdimension scores than those who did not (*p* = 0.001). Their total VAAI scores and shyness subdimension scores were significantly lower than those who did not participate in scientific programmes (*p* = 0.007; *p* = 0.000, respectively). Additionally, the nurses who received assertiveness and leadership training had lower shyness subdimension scores compared to those who did not receive such training (*p* = 0.025). The nurses who perceived themselves as leaders had higher assertiveness subdimension scores than those who did not consider themselves as leaders (*p* = 0.000). Their total VAAI scores and shyness subdimension scores were lower than those who did not perceive themselves as leaders (*p* = 0.034; *p* = 0.000, respectively) (Table 3).

The nurses with a postgraduate degree had lower shyness subdimension scores compared to those with only a bachelor's degree (*p* = 0.010). The total VAAI score was also lower in the nurses with a postgraduate degree (91.51 ± 14.773). However, there was no statistically significant difference in the LOS and VAAI total scores or any subdimensions between those with a vocational high school education and those with an associate degree (*p* > 0.05) (Table 3).

Regarding the distribution of LOS scores based on demographic and professional characteristics, the married nurses had higher structural leadership subdimension scores than the single nurses (*p* = 0.011). The nurses living in the city centre had higher human resource leadership subdimension scores than those living in districts or villages (*p* = 0.002). The nurses who enjoyed their profession had higher total LOS scores and all subdimension scores than those who did not enjoy their profession (*p* = 0.000; *p* = 0.010; *p* = 0.001; *p* = 0.000; *p* = 0.000, respectively). Similarly, the nurses who would choose the nursing profession again had higher total LOS scores and all subdimension scores than those who would not (*p* = 0.000; *p* = 0.000; *p* = 0.001; *p* = 0.000; *p* = 0.000, respectively) (Table 3).

The nurses who participated in scientific programmes had higher total LOS scores and higher subdimension scores compared to those who did not participate (*p* = 0.000; *p* = 0.001; *p* = 0.001; *p* = 0.000; *p* = 0.000, respectively). Similarly, the nurses who received assertiveness and leadership training had higher total LOS scores and all subdimension scores compared to those who did not receive such training (*p* = 0.000; *p* = 0.011; *p* = 0.016; *p* = 0.000; *p* = 0.000, respectively). The nurses who selected a leader nurse as a role model had higher total LOS scores, as well as higher scores in the political leadership and symbolic leadership subdimensions, compared to those who did not (*p* = 0.024; *p* = 0.040; *p* = 0.025, respectively). Moreover, the nurses who perceived themselves as leaders had higher total LOS scores and higher subdimension scores across all dimensions compared to those who did not see themselves as leaders (*p* = 0.000; *p* = 0.000; *p* = 0.000; *p* = 0.000; *p* = 0.000) (Table 3).

A statistically significant difference was observed between those nurses with a bachelor's degree and those with a postgraduate degree in terms of total LOS scores, as well as the Human Resource leadership, Political leadership and Symbolic leadership subdimensions, along with the shyness subdimension scores. The nurses with a postgraduate degree demonstrated higher total LOS scores and subdimension scores compared to their counterparts with a bachelor's degree (*p* = 0.004; *p* = 0.042; *p* = 0.001; *p* = 0.009, respectively) (Table 3).

The mean total score of the VAAI was 97.52 ± 16.031. The mean score for the shyness subdimension was 48.87 ± 17.214, while the mean score for the assertiveness subdimension was 48.65 ± 9.113. The mean total score of the LOS was 124.37 ± 17.099. Among its subdimensions, the mean scores were as follows: Human Resource leadership 34.13 ± 3.827, Structural leadership 32.57 ± 4.081, Political leadership 28.66 ± 5.406 and Symbolic leadership 29.02 ± 5.627 (Table 2).

**TABLE 2:** Distribution of VAAI and LOS subdimension scores.

	Mean ± SD	Min–Max
Leadership Orientations Scale (LOS)		
Subdimensions		
Human resource leadership	34.13 ± 3.827	19–40
Structural leadership	32.57 ± 4.081	14–40
Political leadership	28.66 ± 5.406	10–40
Symbolic leadership	29.02 ± 5.627	8–40
Total score	124.37 ± 17.099	55–160
Voltan‐Acar Assertiveness Inventory (VAAI)		
Subdimensions		
Shyness	48.87 ± 17.214	17–102
Assertiveness	48.65 ± 9.113	11–66
Total score	97.52 ± 16.031	28–168

Abbreviations: Max, maximum; Mean, average; Min, minimum; SD, standard deviation.

When examining the relationships between the LOS and VAAI scores, no significant linear correlation was found between the total LOS and VAAI scores. However, a low to moderate negative correlation was observed between the shyness subdimension of the VAAI and the total LOS score, as well as all LOS subdimensions. A moderate positive correlation was found between the assertiveness subdimension of the VAAI and the total LOS score, as well as all LOS subdimensions (Table 4).

## 4 Discussion

The discussion is structured under two separate subheadings: assertiveness levels and leadership characteristics of nurses.

### 4.1 Discussion of Findings Related to the Voltan‐Acar Assertiveness Inventory

Since a total score between 64 and 98 on the VAAI indicates moderate shyness, and the mean score of the shyness subdimension in this study was found to be higher than the assertiveness subdimension, it can be concluded that the participating nurses exhibited moderate shyness (Table 2). Several factors may contribute to this finding, including the social status and value of nursing in society, the historical evolution of the profession, working conditions and the professionalisation process of nursing. Similar findings were reported in other studies using different assertiveness measurement tools, where nurses were also found to display predominantly shy behaviours (Parlar Kılıç and Sevinç 2018; Rezayat and Dehghan Nayeri 2014).

This study found that female nurses were more assertive than male nurses (Table 3). The reason for this may be that assertiveness, as a social behaviour, is influenced by the cultural elements in which individuals are raised and live. However, the literature presents contradictory findings. While some studies found no gender‐based differences in assertiveness (Polat 2013), others reported that male nurses were more assertive (Şekerci 2021). Some studies also found that female nurses exhibited higher assertiveness levels (Adana et al. 2009). The differences between studies may be attributed to variations in cultural structures, differences in the social environments in which nurses live and the predominance of women in the nursing profession. A significant difference was observed in assertiveness levels across different age groups in this study. Specifically, the nurses aged 29 and below had lower assertiveness subdimension scores compared to those in the 30–41 age group (Table 3). Consequently, it was found that the nurses aged 29 and below exhibited more shy characteristics. This suggests that assertiveness skills improve with age. Considering the characteristics of assertiveness, younger nurses may face challenges in asserting their opinions, defending their rights, saying ‘no’ when necessary, and communicating effectively. A previous study reported different results regarding the relationship between age and assertiveness (Polat 2013). The discrepancies among studies may stem from differences in sociodemographic characteristics and variations in the assertiveness scales used.

**TABLE 3:** Distribution of Leadership Orientations Scale and Voltan‐Acar Assertiveness Inventory Scores by nurses' demographic and professional characteristics (*n* = 367).

Leadership Orientations Scale (LOS)							Voltan‐Acar Assertiveness Inventory (VAAI)		
Demographic variables		Total score	Human resource leadership	Structural leadership	Political leadership	Symbolic leadership	Total score	Shyness	Assertiveness
		Mean ± SD	Mean ± SD	Mean ± SD	Mean ± SD	Mean ± SD	Mean ± SD	Mean ± SD	Mean ± SD
Gender	Female (*n* = 319)	124.43 ± 16.504	34.19 ± 3.651	32.63 ± 3.957	28.63 ± 5.268	28.97 ± 5.543	96.82 ± 15.280	48.16 ± 16.820	48.66 ± 9.227
	Male (*n* = 48)	123.98 ± 20.828	33.69 ± 4.865	32.15 ± 4.851	28.83 ± 6.309	29.31 ± 6.213	102.18 ± 19.901	53.55 ± 19.171	48.63 ± 8.403
	*t; p*	0.170; 0.865	0.855; 0.393	0.766; 0.444	−0.243; 0.809	−0.387; 0.699	−1789; 0.079	−2031; 0.043*	0.018; 0.986
Age group	29 years and below *1 (*n* = 123)	123.52 ± 15.615	33.59 ± 3.580	32.18 ± 3.852	28.78 ± 4.900	28.98 ± 5.351	97.61 ± 16.022	50.84 ± 16.946	46.78 ± 8.648
	30–41 years*2 (*n* = 112)	125.47 ± 18.574	34.29 ± 4.093	32.72 ± 4.352	28.96 ± 5.754	29.50 ± 6.045	96.51 ± 15.053	46.60 ± 16.086	49.91 ± 8.209
	42 years and above*3 (*n* = 132)	124.23 ± 17.197	34.49 ± 3.789	32.80 ± 4.056	28.29 ± 5.569	28.65 ± 5.523	98.29 ± 16.895	48.95 ± 18.251	49.34 ± 10.007
	*F; p*	0.388; 0.679	1951; 0.144	0.844; 0.431	0.509; 0.602	0.693; 0.501	0.378; 0.686	1788; 0.169	4110; 0.017* Difference; 1−2
Marital status	Marries (*n* = 219)	125.30 ± 16.077	34.38 ± 3.635	33.01 ± 3.778	28.69 ± 5.231	29.21 ± 5.333	97.08 ± 15.968	48.01 ± 16.597	49.07 ± 9.177
	Single (*n* = 148)	123.00 ± 18.480	33.75 ± 4.079	31.91 ± 4.425	28.61 ± 5.673	28.73 ± 6.042	98.17 ± 16.156	50.13 ± 18.070	48.04 ± 9.013
	*t; p*	1262; 0.207	1559; 0.120	2545; 0.011*	0.141; 0.888	0.809; 0.419	−0.642; 0.521	−1157; 0.248	1056; 0.292
Place of residence	City centre (*n* = 297)	124.81 ± 16.893	34.42 ± 3.738	32.66 ± 4.092	28.60 ± 5.404	29.13 ± 5.443	96.57 ± 15.424	47.88 ± 17.050	48.68 ± 9.246
	District/Village (*n* = 70)	122.51 ± 17.957	32.89 ± 3.977	32.19 ± 4.041	28.89 ± 5.449	28.56 ± 6.372	101.56 ± 17.946	53.04 ± 17.402	48.53 ± 8.588
	*t; p*	1010; 0.313	3.053; 0.002**	0.868; 0.386	−0.394; 0.694	0.763; 0.446	−2.360; 0.019*	−2.265; 0.024*	0.127; 0.899
Enjoyment of the nursing profession	Yes (*n* = 264)	126.61 ± 16.396	34.44 ± 3.893	33.02 ± 3.959	29.35 ± 4.976	29.80 ± 5.278	96.49 ± 15.271	47.41 ± 16.74	49.08 ± 8.986
	No (*n* = 103)	118.63 ± 17.601	33.34 ± 3.550	31.40 ± 4.176	26.88 ± 6.051	27.01 ± 6.011	100.15 ± 17.639	52.60 ± 17.92	47.55 ± 9.384
	*t; p*	4.102; 0.000***	2.585; 0.010*	3.478; 0.001**	3.678; 0.000***	4.378; 0.000***	−1.973; 0.066	−2.616; 0.009**	1.448; 0.149
Willingness to choose the nursing profession again	Yes (*n* = 124)	128.94 ± 16.609	34.86 ± 3.976	33.52 ± 4.151	30.07 ± 4.846	30.48 ± 5.265	96.55 ± 13.712	46.44 ± 15.451	50.11 ± 8.264
	No (*n* = 243)	122.04 ± 16.908	33.75 ± 3.701	32.08 ± 3.967	27.93 ± 5.542	28.27 ± 5.669	98.01 ± 17.099	50.11 ± 17.951	47.91 ± 9.444
	*t; p*	3.717; 0.000***	2.649; 0.000***	3.224; 0.001**	3.644; 0.000***	3.621; 0.000***	−0.887; 0.376	−1.939; 0.053	2.206; 0.028*
Participation in scientific programmes (Congress. symposium. workshop. etc.)	Yes (*n* = 208)	127.32 ± 17.212	34.69 ± 3.899	33.16 ± 4.017	29.53 ± 5.365	29.94 ± 5.739	95.56 ± 15.903	45.52 ± 17.219	50.05 ± 8.471
	No (*n* = 159)	120.52 ± 16.211	33.40 ± 3.614	31.79 ± 4.047	27.52 ± 5.261	27.81 ± 5.254	100.08 ± 15.885	53.25 ± 16.244	46.83 ± 9.615
	*t*; *p*	3.846; 0.000***	3.244; 0.001**	3.218; 0.001**	3.592; 0.000***	3.655; 0.000***	−2.696; 0.007**	−4.368; 0.000***	3.399; 0.001**
Receiving assertiveness and leadership training	Yes (*n* = 79)	130.38 ± 15.704	35.10 ± 3.608	33.54 ± 3.734	30.59 ± 4.906	31.14 ± 5.195	94.83 ± 14.758	45.02 ± 18.186	49.80 ± 8.592
	No (*n* = 288)	122.72 ± 17.122	33.86 ± 3.848	32.30 ± 4137	28.13 ± 5.423	28.44 ± 5.609	98.26 ± 16.309	49.92 ± 16.817	48.34 ± 9.240
	*t; p*	3.583; 0.000***	2.571; 0.011*	2.419; 0.016*	3.658; 0.000***	3.851; 0.000***	−1.690; 0.092	−2.254; 0.025*	1.270; 0.205
Having a nurse as a leadership role model	Yes (*n* = 141)	126.92 ± 16.722	34.60 ± 3.670	33.09 ± 3.846	29.39 ± 5.390	29.85 ± 5.668	97.21 ± 14.015	47.76 ± 16.518	49.46 ± 8.324
	No (*n* = 226)	122.78 ± 17.176	33.84 ± 3.902	32.24 ± 4.197	28.20 ± 5.377	28.50 ± 5.551	97.71 ± 17.197	49.56 ± 17.635	48.15 ± 9.555
	*t; p*	2.271; 0.024*	1.855; 0.064	1.929; 0.055	2.062; 0.040*	2.250; 0.025*	−0.287; 0.774	−0.976; 0.330	1.383; 0.167
Perceiving oneself as a leader	Yes (*n* = 202)	131.56 ± 14.98	35.39 ± 3.746	33.95 ± 3.625	30.75 ± 4.626	31.47 ± 4.794	95.91 ± 16.241	45.15 ± 17.160	50.76 ± 8.443
	No (*n* = 165)	115.57 ± 15.36	32.58 ± 3.335	30.87 ± 3.977	26.09 ± 5.192	26.02 ± 5.109	99.49 ± 15.594	53.42 ± 16.202	46.07 ± 9.260
	*t*; *p*	10.057; 0.000***	7.505; 0.000***	7.744; 0.000***	9.087; 0.000***	10.501; 0.000***	−2.134; 0.034*	−4.709; 0.000***	5.076; 0.000***
Educational level	1. Vocational health high school	127.80 ± 20.346	34.70 ± 4.508	33.00 ± 4.746	30.25 ± 6.248	29.85 ± 6.235	100.10 ± 23.033	52.19 ± 22.403	47.92 ± 11.109
	2. Associate degree	124.41 ± 13.780	34.10 ± 3.194	32.64 ± 3.328	28.44 ± 4.695	29.23 ± 5.137	97.77 ± 13.891	48.53 ± 14.080	49.24 ± 8.147
	3. Bachelor's degree	122.80 ± 17.252	33.85 ± 3.867	32.29 ± 4.174	28.13 ± 5.405	28.52 ± 5.696	98.24 ± 15.770	49.95 ± 16.997	48.29 ± 8.962
	4. Postgraduate degree	132.67 ± 15.039	35.62 ± 3.506	34.02 ± 3.551	31.43 ± 4.753	31.60 ± 4.659	91.51 ± 14.773	40.73 ± 16.749	50.77 ± 9869
	*F;p*	4.436;0.004**	2.771;0.042*	2.286;0.078	5.298;0.001**	3.889;0.009**	2.351;0.072	3.818;0.010*	1.000;0.393
	Difference;3 − 4								

Abbreviations: *F*, One‐Way Analysis of Variance (ANOVA); Mean, mean; *p*, significance level; SD, standard deviation; *t*, independent sample *T*‐test.

* *p* < 0.050.

** *p* < 0.010.

*** Mean = mean.

The participating nurses who lived in the city centre for the longest duration were found to be more assertive (Table 3). Assertiveness is influenced by environmental conditions, cultural factors and individual characteristics. The way society approaches events and the manner in which individuals express themselves to establish a place in a social environment can impact assertiveness (Zengin and Alver 2017). These factors help explain why those living in city centres exhibited more assertive behaviours. Similarly, Polat (2013) reported that nurses who lived in urban areas for extended periods demonstrated more assertive behaviour. Additionally, studies by Dinçer and Öztunç (2009) found that students residing in metropolitan cities had higher assertiveness levels. These findings align with our study. It is thought that individuals living in metropolitan areas are expected to be more proactive and independent, leading them to develop greater self‐confidence and assertiveness. However, some studies (Güler 2011; Metin 2014). found no significant difference in shyness or assertiveness subdimension scores based on place of residence.

The nurses with postgraduate degrees had lower shyness subdimension scores compared to those with bachelor's degrees (Table 3). Since lower shyness scores indicate higher assertiveness (Voltan‐Acar and Öğretmen 2007), the increase in assertive behaviours with higher education levels can be considered a positive finding. This suggests that nurses with postgraduate education exhibit assertive behaviours such as advocacy, openness to innovation and ease in achieving their goals. A study conducted abroad on the relationship between education level and assertiveness revealed that nurses with a bachelor's degree were more assertive than nurses with a postgraduate degree (Jinks and Bradley 2004). Similarly, our study found that those who enjoyed their profession and those who would choose nursing again if given the opportunity were more assertive (Table 3). The passion of healthcare professionals for their profession plays a crucial role in the advancement and professionalisation of nursing. Thus, this finding is significant for the development of the profession. A study examining the assertiveness levels of hospital nurses found that nurses who willingly chose their profession had higher assertiveness levels (Çakır 2010).

In our study, the nurses who participated in scientific meetings and received assertiveness and leadership training were found to be more assertive (Table 3). Nurses who receive assertiveness training and attend scientific programmes are likely to be more willing to apply what they have learned, more attentive in recognising opportunities and more proactive in problem‐solving. Based on these findings, it is evident that attending scientific meetings and receiving assertiveness and leadership training are crucial for nurses, and it is recommended that nurses be supported in these areas. In the literature, a study conducted with nurses and nursing students also found a significant difference in assertiveness scale scores before and after assertiveness training (Lin et al. 2004).

Our study also revealed that those nurses who perceived themselves as leaders exhibited higher assertiveness levels (Table 3). Assertive individuals are more inclined to demonstrate leadership characteristics, as assertiveness is one of the key traits of modern leadership. Through assertiveness skills, leaders can persuade and influence their followers, take risks for change and innovation and effectively navigate challenges (Barutçugil 2014).

### 4.2 Discussion of Findings Related to the Leadership Orientations Scale

The findings suggest that the nurses who participated in this study exhibited leadership behaviours (Table 2). This can be attributed to the fact that a large proportion of the participating nurses held a bachelor's degree (72.4%) and had 16 or more years of professional experience (46.3%) (Table 1). Additionally, the increasing prevalence of undergraduate and postgraduate education in Türkiye may have contributed to this outcome. Similar findings were reported in other studies, where high total scores on the LOS were observed (Coşkun and Arslan 2021; Gürsoy and Uzun Aksoy 2021).

The study found that the Human Resource Leadership subdimension had the highest mean score compared to the other subdimensions (Table 2). Human Resource Leadership encompasses interpersonal communication, consideration of individuals' needs and desires, active listening and encouraging participation in new ideas (Dereli 2003). It has also been suggested that developing human resource leadership characteristics can facilitate the enhancement of other leadership orientations (Özdemir Özkan et al. 2015). Therefore, the fact that the participating nurses demonstrated Human Resource Leadership characteristics is a significant finding. Given that nursing is inherently a human‐centred profession, where nurses maintain continuous communication with patients, healthy individuals and their families, and where challenges are often human‐related, it is understandable that nurses exhibit Human Resource Leadership behaviours. Additionally, factors such as nurses' intrinsic motivation for their profession, their relatively high average age and their extensive experience may have played a role in this outcome. It is also possible that individuals who naturally exhibit Human Resource Leadership traits are more likely to choose nursing as a profession. Similar findings were reported in previous research (Aksu and Yiğit 2019; Coşkun and Arslan 2021; Gürsoy and Uzun Aksoy 2021; Özdemir Özkan et al. 2015).

In our study, the lowest mean score was observed in the Structural Leaders subdimension (Table 2). One possible explanation for this finding is that data collection occurred during the COVID–19 pandemic, which may have led the participating nurses to overlook change and transformation during that period. Structural Leadership is characterised by leaders who adopt innovations in practice, are willing to embrace change, possess strong influence over others, effectively manage crises and serve as role models for their teams (Dereli 2003; Samsir 2018). The fact that the Structural Leaders subdimension had the lowest mean score suggests that the nurses in our study were hesitant to adopt innovations, avoided taking risks, adhered to traditional approaches in practice and struggled to effectively intervene in challenges. Based on these results, it is evident that nurses need to develop their Structural Leadership skills. Similar findings were reported in other studies, where the lowest scores were also recorded in the Structural Leaders subdimension (Aksu and Yiğit 2019; Coşkun and Arslan 2021; Gürsoy and Uzun Aksoy 2021).

The participating nurses with postgraduate degrees exhibited stronger leadership characteristics compared to those with bachelor's degrees (Table 3). This finding suggests that leadership traits such as assertiveness, decisiveness, consistency, openness to innovation and reliability can be acquired through education. Additionally, it is possible that individuals who already possess leadership qualities may be more inclined to pursue postgraduate education. Considering both possibilities, this result highlights the importance of nursing education in leadership development. Similarly, in a study by Aksu and Yiğit (2019), nurses with undergraduate and postgraduate degrees had higher leadership scores compared to those with lower education levels. The International Council of Nurses (ICN) has also emphasised in recent years that nurses with leadership qualities are essential for ensuring that the profession receives the recognition it deserves (ICN 2022). The transition of nursing education from vocational high school and associate degree levels to undergraduate programmes, as well as the increase in universities offering postgraduate nursing education, has played a crucial role in the development of nurse leaders (T.C. Official Gazette 2007). The study also found that the participating married nurses exhibited stronger Structural Leadership characteristics compared to the single nurses (Table 3). Structural Leadership involves working harmoniously with a team, adapting quickly to the environment and maintaining a structured and organised work approach. These results indicate that marital status may influence leadership orientations. However, in studies examining leadership behaviours among nurse managers, no significant difference was found based on marital status (Arabacı 2012; Soyluer 2010). This discrepancy may be due to the fact that previous studies focused only on nurse managers, who may possess distinct personal characteristics compared to general nursing staff. Additionally, those nurses who lived in city centres exhibited stronger Human Resource Leadership characteristics than those who lived in districts or rural areas (Table 3). Nurses who demonstrate Human Resource Leadership traits tend to prioritise the emotions, thoughts, needs and desires of individuals and effectively utilise empathy skills (Samsir 2018). The intensity of social interactions in metropolitan areas and the interpersonal communication demands of nursing may have contributed to enhancing Human Resource Leadership skills among those who spent more time in urban settings.

The participating nurses who enjoyed their profession exhibited stronger leadership characteristics compared to those who did not enjoy their profession (Table 3). This finding suggests that nurses who are passionate about their profession are more likely to develop leadership skills and act as leaders or potential leaders. It is expected that nurses who enjoy their profession would demonstrate strong Human Resource Leadership characteristics. Given that nursing is a human‐centred profession, nurses who are passionate about their work are likely to build positive relationships, be active listeners, show empathy towards both patients and healthy individuals, understand the needs of their colleagues and perform their duties with a humanistic approach. Additionally, nurses who enjoy their profession also exhibit Structural Leadership characteristics by following regulations, valuing the smooth execution of tasks and taking responsibility for the outcomes of their work. Furthermore, these nurses are more likely to encourage others towards change, implement innovations, take risks and initiate transformation, all of which are key aspects of political leadership (Samsir 2018).

Similarly, those nurses who would choose the nursing profession again demonstrated stronger leadership characteristics than those who would not (Table 3). This finding suggests that individuals with leadership traits are more inclined to choose nursing as a profession. Moreover, choosing nursing again may indicate a deep passion for the profession, further reinforcing leadership tendencies. Nurses who are committed to their profession tend to exhibit structural leadership characteristics, such as monitoring patient care outcomes, taking responsibility and adhering to professional standards. They also display human resource leadership traits, such as valuing the emotions and thoughts of their colleagues and patients and excelling in interpersonal communication. Additionally, nurses who are passionate about their profession are more likely to engage in Political Leadership by taking risks, following and implementing innovations and actively contributing to the advancement of nursing. Lastly, they may exhibit symbolic leadership traits, including crisis management, problem‐solving and analytical thinking. Considering these findings, choosing nursing willingly plays a crucial role in the professional development and advancement of the field (Samsir 2018).

Those nurses who participated in scientific programmes and received assertiveness and leadership training exhibited greater openness to change and a stronger ability to implement innovations compared to those who did not receive such training (Table 3). This finding supports the idea that leadership characteristics can be developed through education and that leadership can be learned. The participating nurses who received education, training or certification in leadership and management demonstrated all leadership orientations and characteristics (Table 3). Actively engaging in scientific programmes, contributing to national and international publications and playing a role in the development of the profession are not only vital for the advancement of nursing but also for the cultivation of nurse leaders. Those nurses who had a leader nurse as a role model exhibited higher Political Leadership and Symbolic Leadership characteristics compared to those who did not have a leader nurse role model (Table 3). Since Political Leaders are risk‐takers who initiate change and adapt easily to new developments, this result suggests that nurses who consider a leader nurse as a role model are more likely to embrace change and transformation (Table 3). Furthermore, those who had a leader nurse as a role model may have been inspired by them, with the leader's behaviours and thought processes serving as a guiding influence. This influence may have contributed to the development of Symbolic Leadership characteristics, which involve charisma, inspiration and vision. The study also found that the nurses who perceived themselves as leaders exhibited all leadership characteristics more strongly than those who did not (Table 3). As in all professions, modern leadership characteristics are crucial in nursing. Therefore, this finding is considered a positive indicator for the future development of the nursing profession. No significant linear relationship was found between the LOS and VAAI total scores among the participating nurses (Table 4). However, relationships were observed between certain subdimensions, suggesting that nurses who exhibit leadership characteristics are expected to demonstrate assertive behaviours. Since a lower shyness subdimension score on the VAAI indicates higher assertiveness, the negative correlation between LOS and shyness is a positive finding. This suggests that as nurses' leadership abilities increase, their shyness decreases. Additionally, as total LOS scores increase, assertiveness increases and shyness decreases. Assertiveness is one of the key characteristics of modern leadership. Individuals who demonstrate assertiveness are also likely to be leaders or potential leaders. In this study, it was observed that as nurses exhibited more leadership characteristics, their assertiveness levels also increased.

**TABLE 4:** Distribution of the relationship between Leadership Orientations Scale Scores and Voltan‐Acar Assertiveness Inventory Scores.

			Voltan‐Acar Assertiveness Inventory		
			Total score	Shyness	Assertiveness
Leadership Orientations Scale	Total score	*r*	−0.035	−0.304	0.513
		*p*	0.504	0.000***	0.000***
	Human resource leadership	*r*	−0.010	−0.274	0.499
		*p*	0.842	0.000***	0.000***
	Structural leadership	*r*	−0.015	−0.293	0.527
		*p*	0.771	0.000***	0.000***
	Political leadership	*r*	−0.059	−0.273	0.411
		*p*	0.256	0.000***	0.000***
	Symbolic leadership	*r*	−0.031	−0.263	0.442
		*p*	0.553	0.000***	0.000***

Abbreviations: *p*, significance level; *r*, Pearson correlation coefficient.

*** *p* < 0.001.

## 5 Limitations

Initially, data collection was planned to be conducted through face‐to‐face methods. However, due to the COVID‐19 pandemic, data collection was shifted to an online format, which constitutes a limitation of this study. Another limitation is that the data were collected using self‐report scales; the assertiveness and leadership characteristics of the nurses were assessed solely based on the items included in these instruments. Therefore, we recommend that future research explore nurses' assertiveness and leadership traits, as well as the factors influencing these characteristics, using qualitative research methods. An additional limitation of this study is the limited availability of the relevant literature. Most existing studies on assertiveness and leadership have been conducted with nurse managers or nursing students, rather than with clinical nursing staff.

## 6 Conclusion and Implications for Practice

This study found that nurses exhibited moderate levels of shyness and above‐average leadership characteristics. Additionally, gender, age, education level, place of residence, passion for the profession, assertiveness training, participation in scientific programmes and self‐perception as a leader positively influenced assertiveness levels. Furthermore, marital status and having a nurse role model as a leader were also identified as factors that positively impacted leadership characteristics.

Based on the study findings, the following recommendations are made:

- In‐service training programmes should be provided to develop charismatic, transformational and structural leadership capacities among nurses.
- Future research should be conducted using diverse measurement tools to allow for a more comprehensive assessment of assertiveness and leadership characteristics.
- The study should be replicated with the inclusion of additional variables that may influence assertiveness and leadership traits.

## Author Contributions

Study design: N.D., A.Ç. Data collection: A.Ç. Data analysis: N.D., A.Ç. Study supervision: N.D. Manuscript writing: N.D., A.Ç. Critical revisions for important intellectual content: A.Ç.

## Funding

The study was supported by a grant from the Eskisehir Osmangazi University Scientific Research Projects Commission [Project number: 201942A115].

## Conflicts of Interest

The authors declare no conflicts of interest.

## Data Availability

The data that support the findings of this study are available from the corresponding author upon reasonable request.

## References

[nop270492-bib-0001] Adana, F. , S. Eliş , B. Aktaş , H. Alkan , S. Erdağ , and Ö. Uluma . 2009. “Determining Assertiveness Level of Student Health Officering and Nursing.” Journal of Atatürk University School of Nursing 12, no. 2: 51–56. https://dergipark.org.tr/tr/pub/ataunihem/issue/2645/34027.

[nop270492-bib-0002] Aksu, D. , and R. Yiğit . 2019. “The Investigation of the Relationship Between the Leadership Traits of Nurses Working in Child Clinics and Family Centered Care.” Adnan Menderes University Faculty of Health Sciences Journal 3, no. 2: 98–110.

[nop270492-bib-0003] Arabacı, S. 2012. “The Influence of Nurses Motivation of the Leadership Behaviours of Nurse Managers Working in Intensive Care Units.” (Thesis number 293114) [Master Thesis, Haliç University]. https://tez.yok.gov.tr/UlusalTezMerkezi.

[nop270492-bib-0004] Ardahan, M. , and E. Konal . 2017. “Management and Leadership in Nursing.” Gümüşhane University Journal of Health Sciences 6, no. 1: 140–147. https://dergipark.org.tr/tr/pub/gumussagbil/issue/32271/358715.

[nop270492-bib-0005] Avcı, E. Ö. , and M. Başer . 2019. “Leadership Characteristics of Nurses in Clinical Decision Making.” Journal of the Faculty of Health Sciences of Erciyes University 6, no. 2: 1–5. https://dergipark.org.tr/en/download/article‐file/940040.

[nop270492-bib-0006] Barutçugil, İ. 2014. Liderlik. 1st ed. Kariyer Publications.

[nop270492-bib-0007] Bayrakdaroğlu, Y. , A. Y. Albayrak , H. Türkay , and M. İ. Kurudirek . 2020. “Investigating Leadership Orientation and Time Management Skills of Basketball Players.” Igdir University Journal of Social Sciences 3, no. 1: 1–12. https://dergipark.org.tr/tr/pub/igdirsbd/issue/57082/792630.

[nop270492-bib-0008] Binuja, P. , and Dr. Nagarajaiah . 2020. “Factors Influencing Assertiveness in Nursing.” International Journal of Scientific and Research Publications 10, no. 3: 829–835. 10.29322/IJSRP.10.03.2020.p99101.

[nop270492-bib-0009] Bolman, L. G. , and T. E. Deal . 1990. “Leadership Orientations.” https://www.scribd.com/document/383760732/Bolman‐L‐G‐Deal‐T‐E‐1990‐Leadership‐Orientations‐Self.

[nop270492-bib-0010] Boone, B. N. , M. L. King , L. S. Gresham , P. Wahl , and E. Suh . 2008. “Conflict Management Training and Nurse‐Physician Collaborative Behaviors.” Journal for Nurses in Staff Development 24, no. 4: 168–175. 10.1097/01.NND.0000320670.56415.91. 18685477

[nop270492-bib-0011] Çakır, A. 2010. “The Relationship Between Nurse's Assertiveness and Self‐Actualization.” (Thesis number 267540) [Master Thesis, Marmara University]. https://tez.yok.gov.tr/UlusalTezMerkezi.

[nop270492-bib-0012] Coşkun, S. , and S. Arslan . 2021. “The Relationship Between Leadership Orientations and Learning Styles of Nursing Students.” Journal of Health and Nursing Management 8, no. 2: 160–170. 10.5222/SHYD.2021.48378.

[nop270492-bib-0013] de Almeida Moura, A. , A. Bernardes , A. P. Balsanelli , C. A. Dessotte , C. S. Gabriel , and A. C. B. Zanetti . 2020. “Leadership and Job Satisfaction in the Mobile Emergency Care Service Context*.” Revista Latino‐Americana de Enfermagem 28: e3260. 10.1590/1518-8345.3455.3260. 32401906 PMC7217629

[nop270492-bib-0014] Dereli, M. 2003. “A Survey Research of Leadership Styles of Elementary School Principals.” (Thesis number 140148) [Master Thesis, METU]. https://tez.yok.gov.tr/UlusalTezMerkezi.

[nop270492-bib-0015] Dinçer, F. , and G. Öztunç . 2009. “Self‐Esteem and Assertiveness Levels of Nursing and Midwifery Students.” Hacettepe University Faculty of Health Sciences Nursing Journal 16, no. 2: 22–33.

[nop270492-bib-0016] Güler, R. 2011. “The Level of Nursing Students Self Esteem and Assertiveness. (Lefkoşa, 2011).” [Master's Thesis, Near East University].

[nop270492-bib-0017] Gürsoy, E. , and M. Uzun Aksoy . 2021. “Determination of Personality Characteristics and Leadership Orientation of Nursing and Midwifery Students.” Journal of Education and Research in Nursing 18, no. 1: 37–43. 10.5152/jern.2021.32744.

[nop270492-bib-0018] International Council of Nurses . 2022. “Investing Nursing and Respecting Nurses Rights Key Themes.” [online]. ICN, Geneve. https://www.icn.ch/news/investing‐nursing‐and‐respecting‐nurses‐rights‐key‐themesinternational‐nurses‐day−2022#:~:text=The%20International%20Council%20o%20Nurses,strengthen%20health%20systems%20around%20the.

[nop270492-bib-0019] Jinks, A. M. , and E. Bradley . 2004. “Angel, Handmaiden, Battleaxe or Whore? A Study Which Examines Changes in Newly Recruited Student Nurses' Attitudes to Gender and Nursing Stereotypes.” Nurse Education Today 24: 121–127. 10.1016/j.nedt.2003.10.011. 14769456

[nop270492-bib-0020] Kuşaklı Yılmaz, B. , and N. Bahçecik . 2012. “The Emotional Intelligence Skills and Leadership Attitudes of Nurse Manager.” Florence Nightingale Journal of Nursing 20, no. 2: 112–119.

[nop270492-bib-0021] Lin, Y. R. , I. S. Shiah , Y. C. Chang , T. J. Lai , K. Y. Wong , and K. R. Chau . 2004. “Evaluation of an Assertiveness Training Program on Nursing and Medical Students' Assertiveness, Self‐Esteem and Interpersonal Communication Satisfaction.” Nurse Education Today 24: 656–665. 10.1016/j.nedt.2004.09.004. 15519449

[nop270492-bib-0022] Manning, M. L. 2006. “Improving Clinical Communication Through Structured Conversation.” Nursing Economics 24, no. 5: 268–271. 17131620

[nop270492-bib-0023] McVanel, S. , and B. Morris . 2010. “Staff's Perceptions of Voluntary Assertiveness Skills Training.” Journal for Nurses in Staff Development 26, no. 6: 256–259. 10.1097/nnd.0b013e31819b5c72. 21119378

[nop270492-bib-0024] Metin, M. 2014. “Assertiveness Levels and Attachment Styles of Nursing Students.” (Thesis number 369936) [Master Thesis, Haliç University]. https://tez.yok.gov.tr/UlusalTezMerkezi.

[nop270492-bib-0025] Miles, J. M. , and E. S. Scott . 2019. “A New Leadership Development Model for Nursing Education.” Journal of Professional Nursing 35, no. 1: 5–11. 10.1016/j.profnurs.2018.09.009. 30709465

[nop270492-bib-0026] Özdemir Özkan, N. , S. Akın , and Z. Durna . 2015. “Nursing Students' Leadership Tendencies and Motivation Levels.” Journal of Education and Research in Nursing 12, no. 1: 51–61. 10.5222/HEAD.2015.051.

[nop270492-bib-0027] Parlar Kılıç, S. , and S. Sevinç . 2018. “The Relationship Between Cultural Sensitivity and Assertiveness in Nursing Students From Turkey.” Journal of Transcultural Nursing 29, no. 4: 379–386. 10.1177/1043659617716518. 28826358

[nop270492-bib-0028] Polat, H. 2013. “Studying of The Relationship Between The Problem Solving Skills of Nurses and Their Assertiveness.” (Thesis Number 376902) [Master Thesis, Adnan Menderes University]. https://tez.yok.gov.tr/UlusalTezMerkezi.

[nop270492-bib-0029] Rezayat, F. , and N. Dehghan Nayeri . 2014. “The Level of Depression and Assertiveness Among Nursing Students.” Journal of Commnunity Based Nursing and Midwifery 2, no. 3: 177–184. https://pmc.ncbi.nlm.nih.gov/articles/PMC4201197/. PMC4201197 25349860

[nop270492-bib-0030] Samsir, S. 2018. “The Effect of Leadership Orientation on Innovation and Its Relationship With Competitive Advantages of Small and Medium Enterprises in Indonesia.” International Journal of Law and Management 60, no. 2: 530–542. 10.1108/IJLMA-01-2017-0005.

[nop270492-bib-0031] Şekerci, Y. 2021. “Relationship Between Cultural Sensitivity and Self‐Determination (Assertiveness) in Nursing Students.” Journal of Education and Research in Nursing 18, no. 1: 24–30. 10.5152/jern.2021.76753.

[nop270492-bib-0032] Serinkan, C. , and İ. İpekçi . 2005. “Leaderhip in Managerial Nurses: A Study for Leadership Characteristics.” Süleyman Demirel University Journal of Faculty of Economics and Administrative Sciences 10, no. 1: 281–294. https://dergipark.org.tr/tr/pub/sduiibfd/issue/20841/223396.

[nop270492-bib-0033] Soyluer, B. 2010. “The Influence of Leadership Behaviours of Nurse Managers on the Motivation of Nurses in Private Hospitals: The Case of Bayindir Hospital.” [Master Thesis, Beykent University]. https://tez.yok.gov.tr/UlusalTezMerkezi.

[nop270492-bib-0034] T.C. Official Gazette . 2007. No: 26510. “Law on Amendment of the Law on Nursing.” https://www.resmigazete.gov.tr/eskiler/2007/05/20070502‐3.htm.

[nop270492-bib-0035] Voltan‐Acar, N. , and T. Öğretmen . 2007. “Self‐Determination (Trustworthiness) Scale Development Studies.” Turkish Psychological Counseling and Guidance Journal 3, no. 27: 67–78. https://dergipark.org.tr/tr/download/article‐file/200088.

[nop270492-bib-0036] World Health Organization . 2020. https://www.icn.ch/sites/default/files/inlinefiles/PR_59_%202020%20International%20Year%20of%20the%20Nurse%20and%20Midwife.pdf.

[nop270492-bib-0037] Zengin, Ö. , and B. Alver . 2017. “Investigation of the Colloge Students Use Their Assertiveness Specifications According to the State Media.” International Journal of Eurasian Education and Culture 2, no. 3: 93–114. https://dergipark.org.tr/tr/pub/ijoeec/issue/33838/374670.

